# Tyrosine-Dependent Phenotype Switching Occurs Early in Many Primary Melanoma Cultures Limiting Their Translational Value

**DOI:** 10.3389/fonc.2021.780654

**Published:** 2021-11-11

**Authors:** Ahmad Najem, Jasper Wouters, Mohammad Krayem, Florian Rambow, Malak Sabbah, François Sales, Ahmad Awada, Stein Aerts, Fabrice Journe, Jean-Christophe Marine, Ghanem E. Ghanem

**Affiliations:** ^1^ Laboratory of Clinical and Experimental Oncology (LOCE), Institut Jules Bordet, Université Libre de Bruxelles, Brussels, Belgium; ^2^ Center for Brain and Disease Research, VIB-KU Leuven, Leuven, Belgium; ^3^ Department of Human Genetics KU Leuven, Leuven, Belgium; ^4^ Center for Cancer Biology, VIB-KU Leuven, Leuven, Belgium; ^5^ Department of Oncology KU Leuven, Leuven, Belgium; ^6^ Department of Surgery, Institut Jules Bordet, Université Libre de Bruxelles, Brussels, Belgium; ^7^ Department of Medical Oncology, Institut Jules Bordet, Université Libre de Bruxelles, Brussels, Belgium; ^8^ Department of Human Anatomy and Experimental Oncology, Université de Mons, Mons, Belgium

**Keywords:** melanoma, primary cultures, phenotype switching, tyrosine, pigmentation

## Abstract

The use of patient-derived primary cell cultures in cancer preclinical assays, including drug screens and genotoxic studies, has increased in recent years. However, their translational value is constrained by several limitations, including variability that can be caused by the culture conditions. Here, we show that the medium composition commonly used to propagate primary melanoma cultures has limited their representability of their tumor of origin and their cellular plasticity, and modified their sensitivity to therapy. Indeed, we established and compared cultures from different melanoma patients propagated in parallel in low-tyrosine (Ham’s F10) or in high-tyrosine (Ham’s F10 supplemented with tyrosine or RPMI1640 or DMEM) media. Tyrosine is the precursor of melanin biosynthesis, a process particularly active in differentiated melanocytes and melanoma cells. Unexpectedly, we found that the high tyrosine concentrations promoted an early phenotypic drift towards either a mesenchymal-like or senescence-like phenotype, and prevented the establishment of cultures of melanoma cells harboring differentiated features, which we show are frequently present in human clinical biopsies. Moreover, the invasive phenotype emerging in these culture conditions appeared irreversible and, as expected, associated with intrinsic resistance to MAPKi. In sharp contrast, differentiated melanoma cell cultures retained their phenotypes upon propagation in low-tyrosine medium, and importantly their phenotypic plasticity, a key hallmark of melanoma cells. Altogether, our findings underline the importance of culturing melanoma cells in low-tyrosine-containing medium in order to preserve their phenotypic identity of origin and cellular plasticity.

## Introduction

Phenotypic plasticity, which allows dynamic transitions between distinct cell states, enables tumor cells to survive under various sub-optimal conditions and rapidly adapt to therapeutics. Melanoma cells are notorious for their high plasticity and ability to switch back and forth between various melanoma cell states, including a (hyper)-differentiated, proliferative/melanocytic and a de-differentiated phenotype, a process highly resemblant to the Epithelial-to-Mesenchymal Transition (EMT) (1, 2). The hyperdifferentiated state, which has so far only be reported in drug-exposed melanoma lesions (3), and the melanocytic one (and to a lesser degree also the intermediate state) are characterized by the expression of the master lineage transcription factor MITF and its downstream melanocytic markers such TYR, TYRP1 and Melan-A/MART1 implicated in cell differentiation and pigment formation (3–7). These cells, just like the cells-of-origin of melanoma, the melanocytes, produce melanin through a process called melanogenesis or pigmentation. On the other hand, the mesenchymal-like state is characterized by the expression of Receptor Tyrosine Kinases (i.e. AXL, EGFR), the ZEB1 transcription factor and genes involved in the WNT5A and TGFβ signaling pathways (3–10). These cells do not longer express MITF, pigmentation genes, and are intrinsically resistant to MAPK inhibition (1, 4, 6, 11). The ability to switch between these different phenotypic states is strongly suspected to enable metastatic dissemination and contribute to therapy resistance (to both targeted therapy and immunotherapy) by allowing melanoma cells to adapt to various microenvironmental cues and stress conditions (1, 6, 12–14).

Tyrosine is the precursor of melanin biosynthesis and the substrate of tyrosinase, a key enzyme of melanogenesis (15–19). It was demonstrated that melanogenesis stimulation promotes oxidative stress and alters mitochondrial respiration and cellular metabolism (20–25). We therefore reasoned that the concentrations of tyrosine in the culture medium may influence melanoma state diversity, cellular plasticity, and, thereby, sensitivity to therapy. Indeed, the most widely and commonly used culture medium to establish melanoma cultures is RPMI 1640. It contains 100 µM of tyrosine, that is about the physiological level found in the blood. At this particular concentration, tyrosine is able to drive pigmentation particularly *in vitro*. Nevertheless, within malignant melanocytes in culture, the generation of eumelanins (solid pigment) from tyrosine occurs in specialized organelles called melanosomes (15–17) in a way that, if they were *in vivo*, would be transferred to the surrounding tissues (e.g keratinocytes). This transfer cannot take place in culture. While some of these melanosomes are released into the culture medium, the remaining accumulates inside the cell. Melanogenesis intermediates include quinonic and indolic species known to be cytotoxic (15), so they contribute to increase intracellular oxidative stress and thus activate survival mechanisms in order to cope with such stress.

For this purpose, we monitored the fate of several melanoma primary cultures harboring different mutations, each derived from a different patient and propagated in parallel in a medium containing low (Ham’s F10) or high (Ham’s F10 supplemented with tyrosine, RPMI 1640 or DMEM) concentrations of tyrosine. We followed an experimental design based on the addition of tyrosine to Ham’s F10 medium to raise its concentration from 10 to 100 µM in order to match the one in the very frequently used RPMI 1640 medium. We then repeated the same comparison with RPMI1640 medium. Of note, although the term - “high tyrosine” is used, it corresponds to the physiological concentration of 100 µM in human blood.

## Materials and Methods

### Effectors and Culture Media

Dasatinib, Pimasertib (AS-703026), Sunitinib, and vemurafenib (RG7204/PLX4032) were from Selleck Chemicals. N-Acetyl-L-cysteine (NAC) and Forskolin (FSK) were from Merck. They were dissolved, according to the manufacturer’s recommendations, aliquoted, and stored at -20°C. Ham’s F10, RPMI 1640 and DMEM culture media were from Lonza.

### Establishment of Melanoma Primary Cultures From Patient Tumor Samples

Samples were obtained from surgical procedures. Tumor tissues were cut to very small pieces, which were incubated overnight in a medium containing dispase, collagenase (Merck), and supplemented with penicillin G, kanamycin sulphate, streptomycin sulphate, and gentamycin at standard concentrations (100μg/ml) (Merck). Then the detached cells were washed and seeded in culture flasks.

Melanoma primary cultures were propagated in the following culture media: Ham’s-F10, Ham’s F10 supplemented with 100μM of tyrosine, RPMI 1640, and DMEM. The media corresponding to each of these conditions were used for the experiments. All these culture media were supplemented with 10% heat-inactivated fetal calf serum (FCS), and with L-glutamine, penicillin, and streptomycin at standard concentrations (Thermo Fisher Scientific) in a humidified air with 5% CO2 at 37°C. Cell culture medium was renewed every 2–3 days. Once the cells were at or near confluence they were subcultured. Melanoma primary cultures were regularly checked for mycoplasma contamination using MycoAlert^®^ Mycoplasma Detection Kit (Lonza).

### Quantification and Removal of Fibroblasts From Primary Cultures

In this study, we used the anti-Cluster Differentiation 90 Monoclonal Antibody (Anti-CD90) to identify and quantify fibroblasts in melanoma primary cultures by flow cytometry (FACS; Beckman Coulter Navios).

Fibroblasts (CD90+ cells) depletion of primary melanoma cultures was performed by applying the MACS magnetic separation system (Miltenyi Biotec). Briefly, cells were magnetically labeled with CD90-MicroBeads and loaded into a MACS^®^ Column (type LD), which was placed in the magnetic field of a MACS Separator. Fibroblasts (CD90+) were retained within the column while melanoma cells (unlabeled cells) run through.

This operation was repeated allowing us to highly enriched the population of human melanoma cells and get rid of fibroblasts.

### Pigmented Cell Detection by Flow Cytometry

We used a method to analyze pigmentation by flow cytometry based on the natural autofluorescence of eumelanins (26, 27). Cells were analyzed by the flow cytometer (Beckman Coulter Navios) using the argon laser (488nm –blue). InfraRed fluorescence was collected using a 750 LP bandpass filter (Beckman Coulter Navios).

### Apoptosis Determination

Apoptotic cells were measured using Annexin V-PE Apoptosis Detection Kit I (Miltenyi Biotec), according to the manufacturer’s recommendations. Briefly, cells were seeded in the corresponding culture medium. One day after plating, the culture medium was replaced by a fresh one and cells were further incubated for 2 days. For detection of apoptosis, cells were collected, centrifuged, washed and resuspended in 100 μl 1× Binding Buffer (Miltenyi Biotec). After addition of 5 μl annexin V-PE, cells were incubated for 15 minutes and then analyzed by flow cytometry (FACS Beckman Coulter Navios).

### RNA-Seq

Total RNA was extracted using the innuPREP RNA mini kit (Analytik Jena), according to the manufacturer’s instructions. After quality assessment using the Bioanalyzer 1,000 DNA chip, RNA-seq libraries were prepared according to the Illumina Truseq stranded mRNA sample preparation guide. Final libraries were pooled and sequenced on a HiSeq4000 (LOCE#1 and #2 in Ham’s F10 and Ham’s F10 with tyrosine; Illumina) and NextSeq500 (LOCE#1 and #2 in Ham’s F10 and RPMI1640; Illumina). RNA-seq reads were cleaned using fastq-mcf (ea-utils) and mapped to the genome (hg19) using STAR. Read counts per gene were obtained from the aligned reads using htseq-count. DESeq2 was used for normalization and differential gene expression analysis (**Table S3**).

### Proliferation Assay

Cell proliferation was assessed by crystal violet assay (28). All cells were seeded in 96-well plates (8x10^3^cells/well). One day after plating, the culture medium was replaced by a fresh one containing effectors or not, depending on experimental conditions, and cells were further cultured for 3 days.

### Cell Migration Assay

Cell migration was assessed using transwell inserts (Corning). Briefly, a total of 1×10^4^ cells in serum-free culture medium were seeded into the upper chamber of a transwell filter with pores of 8 μm. These inserts were placed into 24-well plates. The lower chamber was filled with 800 µl of corresponding culture medium containing 10% FCS. In the case of treatment, cells were incubated in the presence or absence of effectors. Cells were allowed to migrate for 24h. Migrated cells were fixed and stained with crystal violet. Images were taken and analyzed using image J. Data are expressed as means ± SEM of three independent experiments.

### ROS Detection

ROS level in cells was detected using CellROX^®^ Green Flow Cytometry Assay Kit (Thermo Fisher Scientific) according to the manufacturer’s instructions. The intensity of CellROX^®^ reflects the level of ROS. Briefly, cells were seeded and incubated for 24 hours. In the case of treatment, cells were incubated in the absence or the presence of effectors. The CellROX reagent was added to the samples at a final concentration of 5 μM. Then, cells were harvested, washed, and analyzed by flow cytometry (FACS Beckman Coulter Navios). Data are represented as the mean of fluorescence intensity ± SEM of three independent experiments.

### Senescence-Associated β-Galactosidase Activity

Senescence-associated β-galactosidase activity was assessed using the BioVision Senescence Detection Kit (BioVision, Mountain View) (29). Briefly, cells were washed twice with PBS, fixed with the fixative solution for 15 minutes at room temperature, and washed again twice with PBS. Then, the staining Solution Mix containing 1 mg/ml X-gal (5-Bromo-4-chloro-3-indolyl-beta-Dgalactopyranoside) was added, and cells were incubated overnight at 37°C. Cells were observed under a microscope equipped with a color CCD camera for the development of blue staining.

### Quantitative Real-Time PCR

Total RNA was extracted from cultured cells using the Qiagen Rneasy Mini kits. cDNA was synthesized using a standard reverse transcription method (qScript cDNA SuperMix, Quanta Biosciences). qPCR reactions were performed using the SYBR Green PCR Master Mix (Thermo Fisher Scientific). The experiments were performed according to the manufacturer’s instructions using QuantStudio™ 3 Thermo Fisher Scientific Real-Time PCR system. The comparative CT method was used to determine relative gene expression levels for each target gene and 18S was used as an internal control for normalization (18S was the most stable gene among 4 references genes tested). The sequences of the primers used for RTq–PCR are available upon request.

### Western Blot Analysis

Cells were plated in Petri dishes (3x10^6^ cells/dish) in culture medium. One day after plating, the culture medium was replaced by a fresh one and further left for 2 days. Then, cells were exposed or not to effectors for 24 hours. Cells were lysed using a detergent cocktail (Thermo Fisher Scientific) and extracted proteins were analyzed by Western blot (28). Immunodetections were performed using antibodies raised against AXL (C89E7), E-cadherin (24E10), p21 (12D1), c-Kit (D13A2) XP^®^, c-Met (D1C2) XP^®^, EGFR (D38B1) XP^®^, Phospho-Rb (Ser608) (D10F2) and MITF (D5G7V) (all from Cell Signaling Technology and dilution used was 1/1000). p53 (DO-1) (1/200), TYRP1 (AB23) (1/1000) and Melan-A (M2-7C10) (1/200) (all from Santa Cruz Biotechnology, Santa Cruz, CA, USA). Tyrosinase (T311°) (1/200) (Thermo Fisher Scientific/WB; Merck/IHC); β-actin (MAB1501R) (1/5000) (Merck) [details on electrophoresis and immunodetection described previously (30)].

### Immunohistochemistry

IHC was done using antibodies raised against AXL (1/50) (from Cell Signaling Technology), Tyrp1 (1/50) and Melan-A (1/100) (all from Santa Cruz Biotechnology, Santa Cruz, CA, USA) and Tyrosinase (1/50) (Merck) in 7 paraffin-embedded tissues and 2 paraffin-embedded primary cultures pellets (primary culture cytospins). These two melanoma primary cultures were derived from 2 of the four tissues. Staining was performed using ultraView Universal Alkaline Phosphatase Red Detection Kit on a BenchMark Ultra (Ventana; Ventana Medical System). Of note, the red staining developed with Fast Red allowed to easily distinguish the immunostaining from black/brown melanin.

### Statistical Analysis

IC50 values represent the inhibitory concentration producing a 50% reduction of cell growth and were calculated from dose-response curves using GraphPad Prism software (GraphPad Software, La Jolla, CA, USA). All data are expressed as means ± SEM of at least three independent experiments. Statistical significance was measured by Student’s t-test using GraphPad Prism software.

## Results

### Tyrosine Promotes Melanogenesis and an Overall Decrease in Viability of Pigmented Primary Cultures

**Figure 1:**
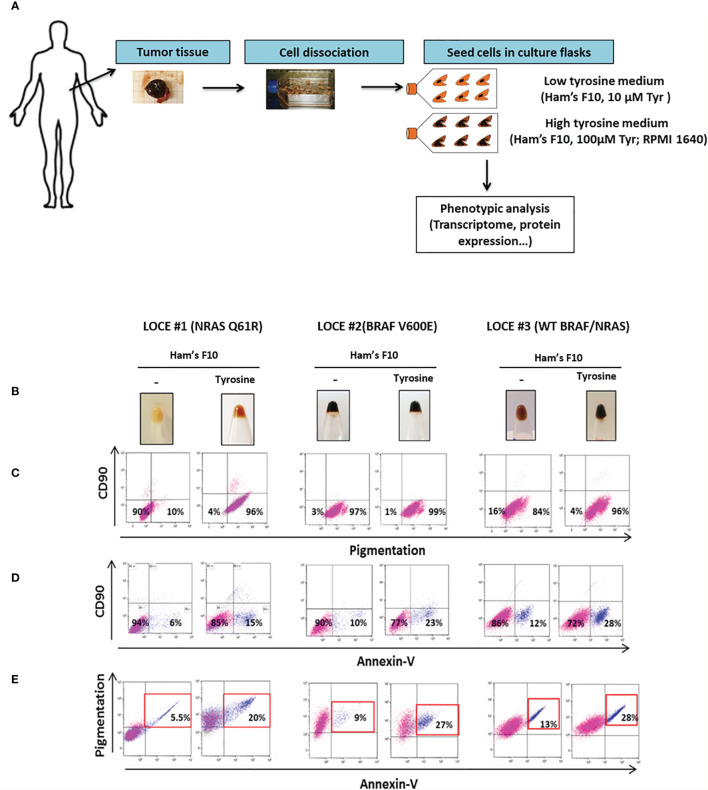
Tyrosine promotes melanogenesis and an overall decrease in viability of pigmented primary cultures. **(A)** Schematic workflow describing the establishment of melanoma primary cultures and downstream phenotypic analysis. Briefly, after surgical dissection; gentle mechanical and enzymatic tissue dissociation processes were used to obtain the single suspension. Then, detached cells were seeded in culture flasks and melanoma primary cultures were propagated in two different microenvironmental conditions with low tyrosine (Ham’s F10) or high tyrosine (Ham’s F10 supplemented with tyrosine, RPMI 1640, or DMEM). **(B)** Monitoring visible pigmentation macroscopically in cell pellets. **(C–E)** Representative flow cytometry plots illustrating (**C)**, pigmentation; **(D)**, cell death (Annexin-V); **(E)**, cell death of pigmented cells in the low tyrosine medium (Ham’s F10) and high tyrosine medium (Ham’s F10, 100µM tyrosine) for 3 representative melanoma primary cultures (LOCE #1, 2 and 3).

**Figure 2:**
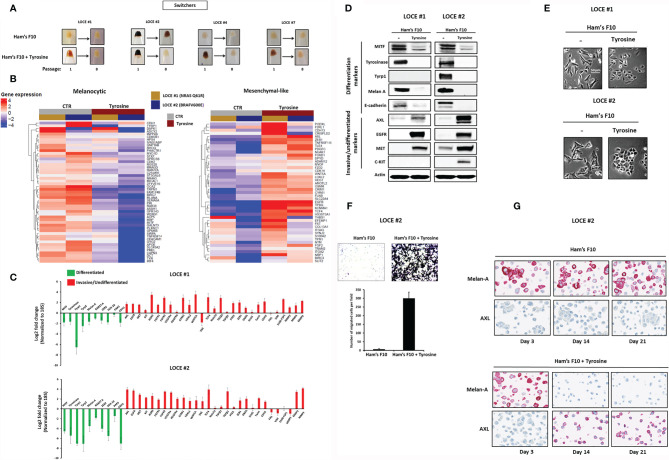
Tyrosine promotes a drift towards a mesenchymal-like/undifferentiated phenotype. **(A)** Comparison of visible pigmentation of cell pellets from four primary cultures (LOCE #1, 2, 4 and 7) (group of switchers) in low tyrosine medium (Ham’s F10, 10 µM) and high tyrosine medium (Ham’s F10, 100 µM/supplemented with tyrosine) at passage 1 and 8. **(B)** Heatmap of genes in the Hoek signatures that are differentially expressed in the primary melanoma cultures (LOCE #1 and 2) at passage 8 for both the melanocytic and mesenchymal-like clusters in the two culture media (Ham’s F10 and Ham’s F10 supplemented with tyrosine) measured by RNA-seq. **(C)** Log2-fold change values determined by Real-time quantitative PCR of main genes implicated in cell differentiation (melanocytic phenotype) (green) and undifferentiation/invasion (mesenchymal-like phenotype) (red) in the primary melanoma cultures (LOCE #1 and 2) (passage 8) (Fold change was calculated based on gene expression in Ham’s F10 high tyrosine relative to Ham’s F10 low tyrosine - Data are presented as Mean ± SEM of three independent experiments normalized to 18S). **(D)** Comparative protein expression (representative western blot) of main differentiation markers and RTK expression (β actin served as a loading control) and **(E)** comparison of cell morphology within the two primary cultures (LOCE #1 and 2) established in the 2 culture media (Ham’s F10 and Ham’s F10 supplemented with tyrosine) (passage 8). **(F)** Effect of tyrosine on cell migration activity in the primary melanoma culture LOCE #2. Upper panel show representative regions of the chamber filters with crystal violet-stained cells. The number of migrated cells per field was calculated from three independent experiments. Data are presented as means ± SEM. **(G)** Immunostaining for melan-A and AXL in low tyrosine medium and high tyrosine medium at day 3, 14 and 21 days in the primary melanoma culture LOCE #2 (magnification, 40).

**Figure 3:**
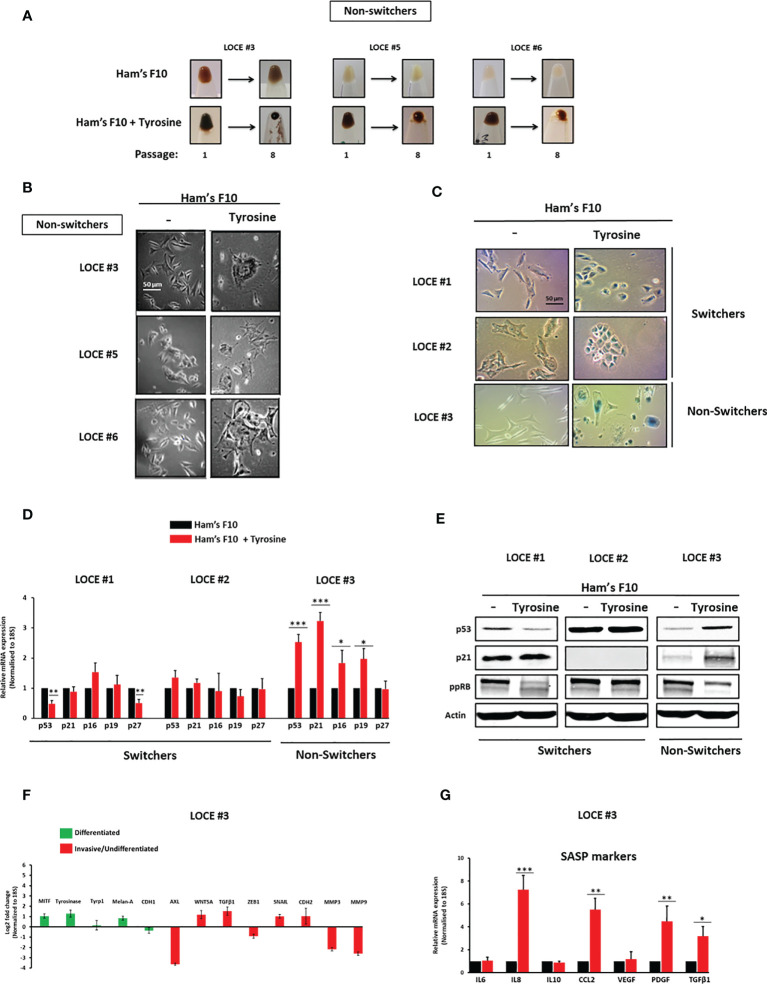
Tyrosine promotes a senescence-like phenotype. **(A)** Comparison of visible pigmentation of cell pellets from three primary cultures (LOCE #3, 5, and 6) (group of non-switchers) in low tyrosine medium (Ham’s F10, 10 µM) and high tyrosine medium (Ham’s F10, 100 µM) at passage 1 and 8. **(B)** Comparison of cell morphology within the non-switchers group in the two culture media (Ham’s F10 and Ham’s F10 supplemented with tyrosine) (passage 8). **(C)**, Comparison of β-Gal activity by *in situ* staining between the primary melanoma cultures (LOCE #1 and 2) (switchers) and the non-switcher primary culture (LOCE #3) in the two culture media (Ham’s F10 and Ham’s F10 supplemented with tyrosine) (passage 8). **(D)** Relative mRNA expression (p53, p21, p16, p27, and p29) and **(E)** protein expression (p53, p21, and ppRB) (representative western blot) of main markers of cell cycle arrest in switchers (LOCE#1 and 2) and non-switcher (LOCE# 3) primary cultures in the two culture media (Ham’s F10 and Ham’s F10 supplemented with tyrosine) (passage 8). **(F, G)** Relative mRNA expression by Real-time quantitative PCR of **(F)** main differentiated (green), invasive markers (red) (Log2-fold change), and **(G)**, SASP (senescence-associated secretory phenotype) markers in non-switcher primary culture (LOCE# 3) (passage 8) (Fold change was calculated based on gene expression in Ham’s F10 supplemented with tyrosine relative to Ham’s F10). Data are presented as means ± SEM from three independent experiments (*p < 0.05; **p < 0.01; ***p < 0.001, t-test).

We established eleven new melanoma primary cultures from metastatic lesions harboring different oncogenic BRAF and NRAS driver mutations (**Table 1**), all explanted in both Ham’s F10 culture medium with 10 or 100 μM tyrosine (**Figure 1A**). Ham’s F10 is a culture medium containing low concentration of tyrosine (10 µM), as opposed to RPMI which contains 100 µM tyrosine. Strikingly, the addition of tyrosine induced/promoted pigmentation in seven of the eleven primary cultures (**Table 1** and **Figures 1B**, **2A**, **3A**). We next evaluated the effect of tyrosine on pigmentation and cell viability by flow cytometry in 3 representative melanoma primary cultures. LOCE #1 is a primary line with NRAS mutation (NRAS Q61R), LOCE #2 is a primary line with BRAF mutation (BRAF V600E) and LOCE #3 is a primary line wild type for both NRAS and BRAF. As anticipated, the tyrosine-dependent induction of pigmentation, which was visible macroscopically in cell pellets (**Figure 1B**), was confirmed by flow cytometry (**Figure 1C**). CD90 was used to monitor the level of fibroblast contamination through the first passages, which was very low or absent (**Figure 1C**). Notably, the induction of pigmentation was associated with a 2-fold increase in cell death, with percentages of apoptotic cells ranging from 15 to 28% (**Figure 1D**). Most dead/dying cells were those exhibiting high pigmentation as illustrated in **Figure 1E**.

**Table 1:** List of melanoma primary cultures used in the study and their classification.

		Primary cultures	BRAF/NRAS mutation status	Visible pigmentation (Cell pellets, P1-P3)	
				Low Tyrosine(Ham’s F10, 10 µM)	High Tyrosine (Ham’s F10, 100 µM or RPMI 1640)
**Pigmented primary cultures**	**Switchers**	LOCE #1	NRAS Q61R	–	+
		LOCE #2	BRAF V600E	+	+
		LOCE #4	BRAF V600E	–	+
		LOCE #7	NRAS Q61R	–	+
	**Non-Switchers**	LOCE #3	WT	+	+
		LOCE #5	NRAS Q61R	–	+
		LOCE #6	BRAF V600E	–	+
**Unpigmented** **primary cultures**		LOCE #8	NRAS Q61K	–	–
		LOCE #9	BRAF V600K	–	–
		LOCE #10	NRAS Q61R	–	–
		LOCE #11	BRAF V600E	–	–

Elven primary melanoma cultures with different mutation status of BRAF and NRAS were established. Seven out of elven cultures were pigmented at first passages (Macroscopic observation of cell pellets) in high tyrosine medium (Ham’s F10, 100µM tyrosine) or/and low tyrosine medium (Ham’s F10) (pigmented primary cultures). In this group, we identified two subgroups the switchers (LOCE #1, 2, 4 and 7) and the non-switchers (LOCE #3, 5, and 6). A distinct group of primary cultures (unpigmented primary cultures) were unable to produce eumelanins (pigmented phenotype) in low or high tyrosine media (LOCE #8, 9, 10, and 11).

### Tyrosine Promotes a Drift Towards a Mesenchymal-Like/Undifferentiated Phenotype

Among pigmented primary cultures (n=7), we identified two mechanisms of cell adaptation following the stress induced *via* the continuous stimulation of melanogenesis by high concentration of tyrosine, which led us to classify primary melanoma cultures into two groups that we termed “switchers” (4/7) (LOCE #1, LOCE #2, LOCE #4 and LOCE #7) and “non-switchers” (3/7) (LOCE #3, LOCE #5, and LOCE #6) (**Table 1**).

In the switchers group, visible pigmentation faded away after only eight passages reflecting a drift from the differentiated (pigment-producing phenotype) towards a de-differentiated phenotype (**Figure 2A**). We profiled NRAS mutant (LOCE #1) and BRAF mutant (LOCE #2) primary cultures at passage eight by RNA-seq in both low and high tyrosine media and found that the level of the Hoek and Verfaillie melanocytic gene expression signatures (4, 31) decreased in high vs low tyrosine culture medium, along with an induction of the undifferentiated/mesenchymal-like gene expression program (**Figure 2B**). Real-time quantitative PCR confirmed the downregulation of the main differentiated/melanocytic markers including MITF and its downstream target genes (TYR, TYRP1, TYRP2/DCT, RAB27A and MLANA) and the up-regulation of the main invasive/undifferentiated markers including RTKs and EMT-TFs such as AXL, EGFR, ZEB1, WNT5A, TGFβ, SNAI1, and TWIST (**Figure 2C**).

Furthermore, this drift was associated with a loss of E-cadherin (CDH1), gene expression and protein levels, a hallmark of EMT induction (**Figures 2B**–**D**), and an up-regulation of MMPs (matrix metalloproteinases) (**Figures 2B, C**). These observations on the transcriptome level were corroborated on the protein level as we observed an inhibition of melanocytic differentiation markers (Tyrosinase, Tyrp1 and Melan-A) associated with an upregulation of RTKs key drivers of the phenotype switching towards an invasive state (AXL (coming from the Greek word “anexelekto”, means uncontrolled), EGFR (Epidermal Growth Factor Receptor), MET (mesenchymal-epithelial transition factor) and C-KIT [referred to as stem cell factor receptor or CD117)](**Figure 2D**). This is consistent with previous transcriptomic data showing that MITF and RTKs (i.e. AXL and EGFR) are parts of two opposing gene expression programs (31–35). Interestingly, we showed that the tyrosine-induced transcriptome reprogramming is phenotypically accompanied with morphological changes from a triangular dendritic morphology (differentiated cells) towards a small round cells morphology (undifferentiated cells) (**Figure 2E**) as well as a dramatic increase in cell migration activity (**Figure 2F**).

To monitor the dynamics of tyrosine-induced phenotype switching, we performed an immunostaining for the most discriminative markers of the differentiated state (Melan-A/MART-1) and invasive/undifferentiated state (AXL). A two week tyrosine challenge was sufficient to cause a profound repression of Melan-A along with a very strong induction of AXL expression (**Figure 2G**). These data show that tyrosine-induced phenotype switching occurs rapidly, only after a few passages. Importantly, AXL-positive cells could not be detected in the cultures before exposure to high tyrosine indicating that the emergence of mesenchymal-like state results from cellular reprogramming rather than a selection of pre-existing cells.

Taken together, our data show that in a subset of melanoma cultures (switchers), a high tyrosine level promotes a phenotype switch from a differentiated to an invasive/undifferentiated state (EMT-like state) associated with MITF repression, EMT-TF reprogramming and RTK induction.

### Tyrosine Also Promotes a Senescence-Like Phenotype

Tyrosine-exposure in the second group of primary cultures (LOCE #3, LOCE #5, and LOCE #6), the non-switchers, resulted in persister cells that showed morphological characteristics of cellular senescence (flat and enlarged morphology) (**Figures 3A, B**).

While in the switchers group (LOCE #1 and LOCE #2) cells exposed to tyrosine drifted to an undifferentiated phenotype, no evidence of senescence-associated β-galactosidase (SA-β-Gal) could be detected (**Figure 3C**). In contrast, cells from the non-switcher group (LOCE #3) exhibited a senescence-like phenotype associated with high SA-β-Gal activity (**Figure 3C**). This was accompanied by a drastic increase in markers of cell cycle arrest at both mRNA (p53, p21, p16 and p19) and protein levels (p53, p21 and decreased ppRB) (**Figures 3D, E**) and inhibition of mesenchymal-like markers (**Figure 3F**).

Senescent cells often release a variety of pro-inflammatory cytokines and growth factors and thus exhibit a senescence-associated secretory phenotype (SASP) (29, 36). Consistent with their increased SA-β-Gal activity, the elevated expression of several SASP associated markers such as IL8, CCL2, PDGF, and TGFβ1 could be detected in the “non-switcher primary culture (LOCE #3) (**Figure 3G**). Together, these results demonstrate that in another subset of melanoma cultures (non-switchers) high tyrosine levels promote a senescence-like phenotype associated with SA-β-Gal activity, cell cycle arrest markers, and activation of SASP.

### ROS Are Drivers of Tyrosine-Induced Phenotypic Switching

**Figure 4:**
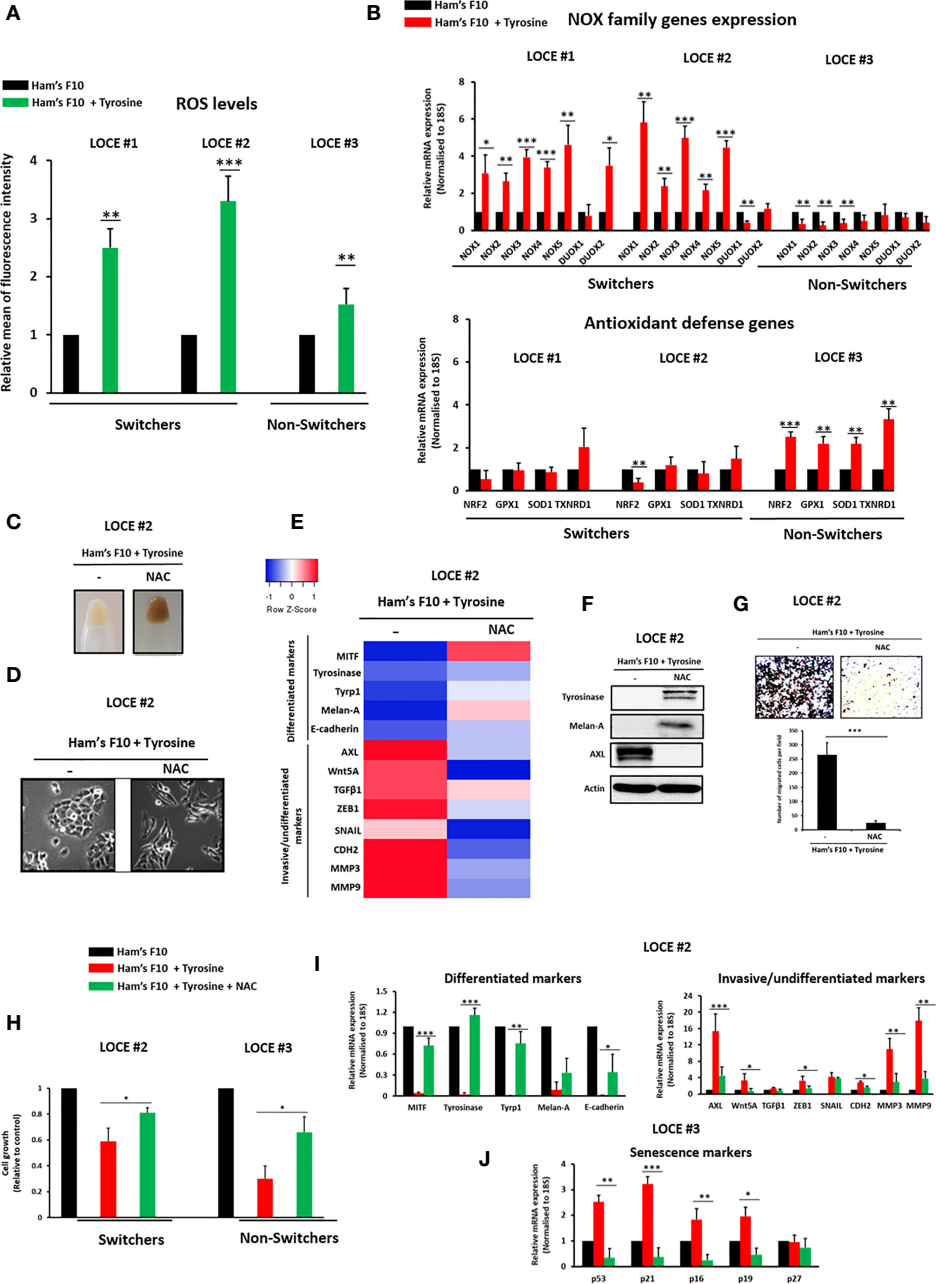
ROS are driver of tyrosine-induced phenotypic switching. **(A)** ROS levels were measured using CellROX-Green flow cytometry assay in the switcher primary melanoma cultures (LOCE #1 and 2) and the non-switcher primary culture (LOCE #3) in the two culture media (Ham’s F10 and Ham’s F10 supplemented with tyrosine). Relative ROS levels are plotted. **(B)** Relative mRNA expression by Real-time quantitative PCR of the NOX family (NOX1-NOX5; DUOX1 and 2) and main anti-oxidant defense genes (NRF2, GPX1, SOD1, and TXNRD1) in switcher primary cultures (LOCE #1 and 2) and non-switcher primary culture (LOCE #3) in the two culture media (Ham’s F10 and Ham’s F10 supplemented with tyrosine) (Fold change was calculated based on gene expression in Ham’s F10 supplemented with tyrosine relative to Ham’s F10 and normalized to 18S). **(C–G)** Restoring the differentiated phenotype by ROS scavenger N-Acetyl-L-cysteine (NAC) in primary culture established in high tyrosine medium (below passage 8). **(C)**, Comparison of visible pigmentation of cell pellets and **(D)**, cell morphology within the switcher primary culture (LOCE #2 in high-tyrosine medium) after 14 days with or without 3 mM NAC. **(E, F) (E)** Heatmap of gene expression levels (average signature Z scores) for representative markers of the differentiated and invasive states (rows) and **(F)** protein expression (representative western blot) of the most discriminative markers of the differentiated (tyrosinase and Melan-A) and the invasive (AXL) phenotypes within the switcher primary culture (LOCE # 2 in high-tyrosine medium) after 14 days with or without 3 mM NAC (columns). **(G)** Effect of NAC on cell migration activity induced by tyrosine in primary culture (LOCE #2 in high-tyrosine medium). Upper panel show representative regions of the chamber filters with crystal violet-stained cells. The number of migrated cells per field was calculated from three independent experiments. **(H–J)** Prevention of phenotypic switch using ROS scavenger NAC (from passage 1). **(H)** Relative cell growth within switcher primary culture (LOCE #2) and non-switcher primary culture (LOCE #3) propagated in culture medium with low tyrosine (Ham’s F10) or high tyrosine (Ham’s F10+ tyrosine) in the presence or the absence of NAC after 14 days. **(I)** Relative mRNA expression of main differentiated (green), invasive markers (red) days within switcher primary culture (LOCE #2) propagated in culture medium with low tyrosine (Ham’s F10) or high tyrosine (Ham’s F10+ tyrosine) in the presence or the absence of NAC after 14 days. **(J)** Relative mRNA expression of main senescence markers within the non-switcher primary culture (LOCE #3) propagated in culture medium with low tyrosine (Ham’s F10) or high tyrosine (Ham’s F10+ tyrosine) in the presence or the absence of NAC after 14 days (Fold change was calculated based on gene expression in Ham’s F10 supplemented with tyrosine relative to Ham’s F10). Data are presented as means ± SEM from from three independent experiments (*p < 0.05; **p < 0.01; ***p < 0.001, t-test).

Reactive Oxygen Species (ROS) were previously shown to promote a phenotypic switch towards an invasive/mesenchymal phenotype in melanoma and colorectal cancer *via* MMP expression (37–39). ROS can be generated by mitochondria, NAPDH oxidase (NOX) protein (40) and melanosomes. Excessive pigment production was indeed shown to induce ROS levels and alter mitochondrial respiration (20–24). We therefore investigated the role of NOX and ROS production in the phenotypic switch induced by tyrosine. We evaluated ROS levels in the switchers (LOCE #1 and LOCE #2) and the non-switcher (LOCE #3). ROS production was higher in switchers than non-switcher cells (**Figure 4A**). We also observe a significant induction of NOX1-NOX5 within the switchers, while no significant changes were observed in antioxidant defense genes (NRF2, GPX1, SOD1, and TXNRD1) (**Figure 4B**). In contrast, NOX expression was not induced in the non-switchers (LOCE #3), while an up-regulation of the anti-oxidant genes was observed (**Figure 4B**).

These data were consistent with a role for ROS as key actor of the tyrosine-induced phenotype switch. Indeed, exposure of the switchers to the ROS scavenger NAC (N-acetylcysteine) was sufficient to prevent a drift towards the de-differentiated phenotype. While NAC restored the pigmented phenotype (**Figure 4C**), the differentiated (dendritic) morphology (**Figure 4D**) and the expression of the melanocytic differentiation markers (**Figures 4E, F**), it inhibited the induction of invasive markers (EMT markers) (**Figures 4E, F**) and the migratory phenotype (**Figure 4G**).

Notably, the reduction in cell viability observed upon tyrosine exposure in both switchers and non-switchers was markedly attenuated by NAC (**Figure 4H**). Moreover, NAC also inhibited the emergence of the invasive phenotype in switchers (**Figure 4I**) and the onset of the senescence-like phenotype in non-switchers (**Figure 4J**). Collectively, these results highlight a critical role for ROS in the modulation of melanoma cell phenotype induced by tyrosine.

### Tyrosine-Induced De-Differentiation Is Reverted by Tyrosine Kinase Inhibitors

**Figure 5:**
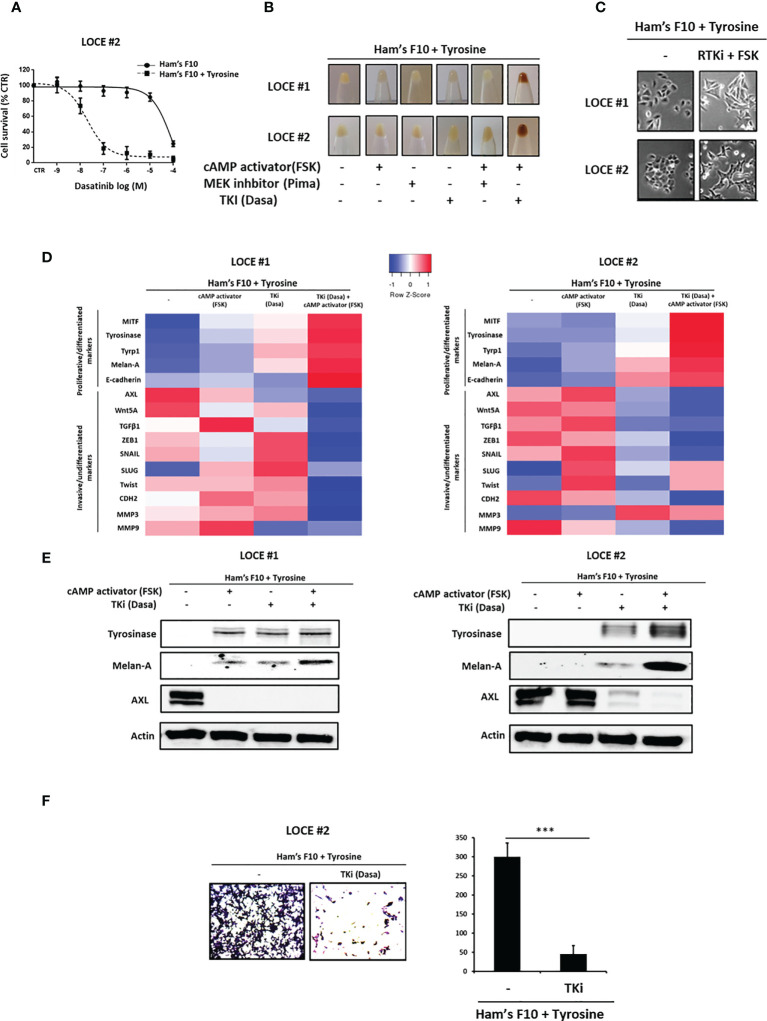
Tyrosine-induced de-differentiation is reverted by Tyrosine kinase inhibitors (TKI). **(A)** Phenotype switching induced by tyrosine leads to marked sensitivity to TKI as showed in survival curves of switcher primary culture (LOCE #2) treated with Dasatinib for 72h and cultured in low versus high tyrosine concentrations. Data are shown as means Mean ± SEM (n=3) compared to untreated cells (control, CTR). **(B–E)** Restoring the pigmented phenotype. **(B)** Comparison of visible pigmentation of cell pellets from the two switcher primary cultures (LOCE #1 and 2) established in high tyrosine medium (Ham’s F10 supplemented with tyrosine) following 14 days after trying to restore pigmented phenotype in this medium by adding a cAMP activator (Forskolin) and different MAPK targeted agents (MEK or TKI inhibitors). **(C)** Comparison of cell morphology in high tyrosine medium (Ham’s F10 supplemented with tyrosine) in the presence or not of an RTK inhibitor (Dasatinib) and cAMP activator (Forskolin) for 14 days. **(D, E) (D)** Heatmap of gene expression levels (average signature Z scores) for representative genes of the differentiated and invasive states (rows) and **(E)** protein expression (representative western blot) of the most discriminative markers of the differentiated (tyrosinase and melanA) and the invasive (AXL) phenotypes within the switcher primary cultures (LOCE # 1 + tyrosine and LOCE # 2 + tyrosine) established in high tyrosine medium after 14 days with an RTK inhibitor (Dasatinib), cAMP activator (Forskolin) either alone or in combination. **(F)** Effect of TKI (Dasatinib) on cell migration activity induced by tyrosine in primary culture (LOCE #2 + tyrosine). left panel shows representative regions of the chamber filters with crystal violet-stained cells. Right panel indicates the number of migrated cells per field calculated from three independent experiments. Data are presented as Mean ± SEM, n=3 (***p < 0.001, t-test).

Next, we tested whether the tyrosine-induced phenotype switching affected the response to a BRAF inhibitor (Vemurafenib), MEK inhibitor (Pimasertib), and tyrosine kinase inhibitors (TKI: Dasatinib and Sunitinib). As expected, once reprogrammed into a mesenchymal-like phenotype melanoma cells became less sensitive to MAPK inhibitors (**Table 2**). More importantly, we found that phenotype switching induced by tyrosine was associated with a marked increase in the sensitivity to TKI (**Table 2** and **Figure 5A**). Indeed, the enhanced sensitivity to TKI could be explained by the induction of RTK expression such as EGFR, AXL, KIT, and MET. These data are consistent with previous studies showing that melanoma cells that adapt to MAPKi through phenotype switching markedly up-regulate several RTK (6, 9, 35, 41).

**Table 2:** Phenotype switching and drug sensitivity.

IC50 values (in micromolar)					
Primary cultures	Culture medium	BRAF inhibitor	MEK inhibitor	Tyrosine kinase inhibitors	
		Vemurafenib	Pimasertib	Dasatinib	Sunitinib
LOCE #1 (NRAS Q61R)	Ham’s F10	17.8	0.001	3.6	9
	Ham’s F10 + Tyrosine	23.2	0.002	1.2	2.2
LOCE #2 (BRAF V600E)	Ham’s F10	3.7	6.1	79.7	27.5
	Ham’s F10 + Tyrosine	16.1	17.1	0.02	1.05

IC50 mean values obtained with BRAFi (Vemurafenib), MEKi (Pimasertib), and TKI (Dasatinib, Sunitinib) with the 2 primary melanoma cultures (switchers: LOCE # 1 and 2) in the 2 culture media (Ham’s F10 and Ham’s F10 supplemented with tyrosine).

We then asked whether the tyrosine-induced mesenchymal phenotype can be reversed and the expression of differentiation markers restored upon exposure to MAPKi (MEKi) and/or cAMP activator (FSK), which are known to enhance the expression of differentiation markers (42, 43). We also evaluated the effect of a TKI, given that the expression of MITF is inversely correlated with the expression of RTK such as AXL and EGFR (32, 35). We show that only the combination of TKI and cAMP activator was able to restore the pigment-producing phenotype (**Figure 5B**) and the differentiated morphology (reverse switch back from small round cells towards the initial triangular dendritic morphology) (**Figure 5C**). Of note, TK inhibitor alone was sufficient to inhibit the expression of invasive markers and restore expression of differentiation markers but this effect was enhanced when combined with a cAMP activator (**Figures 5D, E**). Moreover, we demonstrated that RTKi could also inhibit the cell migration phenotype (**Figure 5F**).

These data suggest that RTK inhibition is sufficient to suppress the mesenchymal-like program and promote differentiation through a mesenchymal-like-to-melanocytic switch.

### Unpigmented Primary Cultures Have an Inherent Invasive Phenotype, Display Intrinsic Resistance to MAPK Inhibitors and Increased Vulnerability to TKI

**Figure 6:**
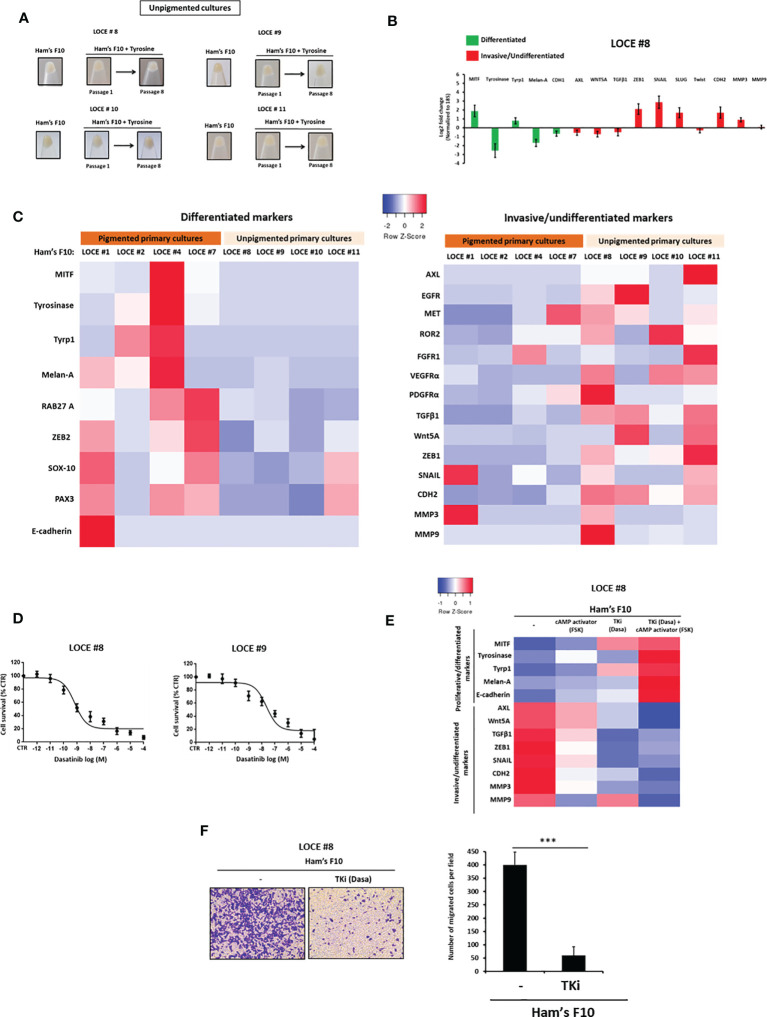
Unpigmented primary cultures have an inherent invasive phenotype, display intrinsic resistance to MAPK inhibitors and increased vulnerability to TKI. **(A)** Comparison of visible pigmentation of cell pellets from four primary cultures (LOCE #8, 9, 10, and 11) (unpigmented cultures) before and following exposure to high concentration of tyrosine at passages 1 and 8. **(B)** Log2-fold change values determined by RTqPCR of main genes implicated in cell differentiation (melanocytic phenotype) (green) and undifferentiation/invasion (mesenchymal-like phenotype) (red) (passage 8) in unpigmented culture (LOCE #8) (Fold change was calculated based on gene expression in Ham’s F10 supplemented with tyrosine relative to Ham’s F10) (passage 8). **(C)** Heatmap of gene expression levels (average signature Z scores) for representative markers of the differentiated and invasive states (rows) in four pigmented (LOCE#1, 2, 4 and 7) and four unpigmented primary cultures (LOCE #8, 9, 10 and 11) (columns) propagated in culture medium with low tyrosine (Ham’s F10). **(D)** Unpigmented primary cultures display marked sensitivity to TKI (dasatinib) as showed in survival curves of unpigmented NRAS mutant primary culture (LOCE #8) and unpigmented BRAF mutant primary culture titrated with TKI (dasatinib) for 72h. Data are shown as Means ± SEM (n=3) compared to untreated cells (control, CTR). **(E)** Heatmap of gene expression levels (average signature Z scores) for representative markers of the differentiated and invasive states (rows) in the unpigmented culture (LOCE #8) after 14 days with an RTK inhibitor (Dasatinib), cAMP activator (Forskolin) either alone or in combination. **(F)** Effect of TKI (Dasatinib) on cell migration activity in unpigmented primary culture (LOCE #8). left panel show representative regions of the chamber filters with crystal violet-stained cells. right panel plots the number of migrated cells per field calculated from three independent experiments. Data are represented as Mans ± SEM from three independent experiments (***p < 0.001, t-test).

A distinct group of primary cultures were unable to produce eumelanins (pigmented phenotype) under high tyrosine challenge (LOCE #8, LOCE #9, LOCE #10 and LOCE #11) (**Table 1**). Macroscopic observation of cell pellets showed a constant and very low level of cell pigmentation upon passages and there is no change in main phenotype markers (**Figures 6A, B**). Accordingly, these unpigmented primary cultures displayed an inherent/innate invasive/mesenchymal-like phenotype (**Figure 6C**). This is further supported by low levels of expression of SOX10, MITF, and downstream differentiation markers such as tyrosinase, TYRP1 and Melan-A. Conversely, these cells expressed high levels of RTKs such as AXL, EGFR, and EMT markers such as ZEB1 and TGFβ1 (**Figure 6C**). Consistently, the expression of melanocytic differentiation markers was very low or almost absent in the corresponding patient’s tumor tissues from which the unpigmented primary melanoma cultures were derived (**Supplementary Figure 1C**). Of note, we did not detect evidence of increased cell death during the establishment of these cultures.

We next evaluated the effect of MAPK inhibitors (BRAFi and MEKi) and TKI in these unpigmented cultures (LOCE #8, LOCE #9, LOCE #10 and LOCE #11) and found them to be all intrinsically resistant to MAPK inhibitors, irrespective of their BRAF and NRAS mutational status (**Table 3**). In contrast, these unpigmented cultures displayed high sensitivity to TKI (**Table 3** and **Figure 6D**), as predicted by their RTK-high gene expression signature.

**Table 3:** Unpigmented primary cultures display an intrinsic resistance to MAPK inhibitors and vulnerability to TKI.

IC50 values (in micromolar)				
Unpigmented cultures	BRAF inhibitor	MEK inhibitor	Tyrosine kinase inhibitors	
	Vemurafenib	Pimasertib	Dasatinib	Sunitinib
LOCE #8 (NRAS Q61K)	45.9	0.006	0.0007	11.7
LOCE #9 (BRAF V600E)	26.8	13.3	0.02	7.1
LOCE #10 (NRAS Q61R)	22.6	0.004	0.0005	2.6
LOCE #11 (BRAF V600E)	13.1	1.8	0.001	5.5

IC50 mean values obtained with BRAFi (Vemurafenib), MEKi (Pimasertib), and TKI (Sunitinib, Dasatinib) in the four unpigmented primary cultures (LOCE #8, LOCE #9, LOCE #10 and LOCE #11) propagated in culture medium with low tyrosine (Ham’s F10).

We also observed that exposure of these unpigmented primary cultures to RTKi (TKI) enhanced the expression of differentiation markers (e.g., Melan-A, tyrosinase) while inhibiting expression of invasive ones (e.g., AXL, MMP3, and MMP9), and this effect was enhanced by cAMP activation (**Figure 6E**). Consistently, RTKi (TKI) also inhibited the ability of these cells to invade *in vitro* (**Figure 6F**).

Altogether, these data indicated that, when grown in low or high tyrosine concentration medium, unpigmented primary cultures maintained their invasive phenotype upon passages, but retained their ability to differentiate following exposure to RTKi.

### Culture Media Composition Influences the Sensitivity to Targeted Drugs

The newly established primary melanoma cultures were grown in parallel in both RPMI 1640, a medium widely used for melanoma cell culture that contains a high concentration of tyrosine (100 μM), and Ham’s F10 medium, which contains a low concentration of tyrosine (10 µM).

**Figure 7:**
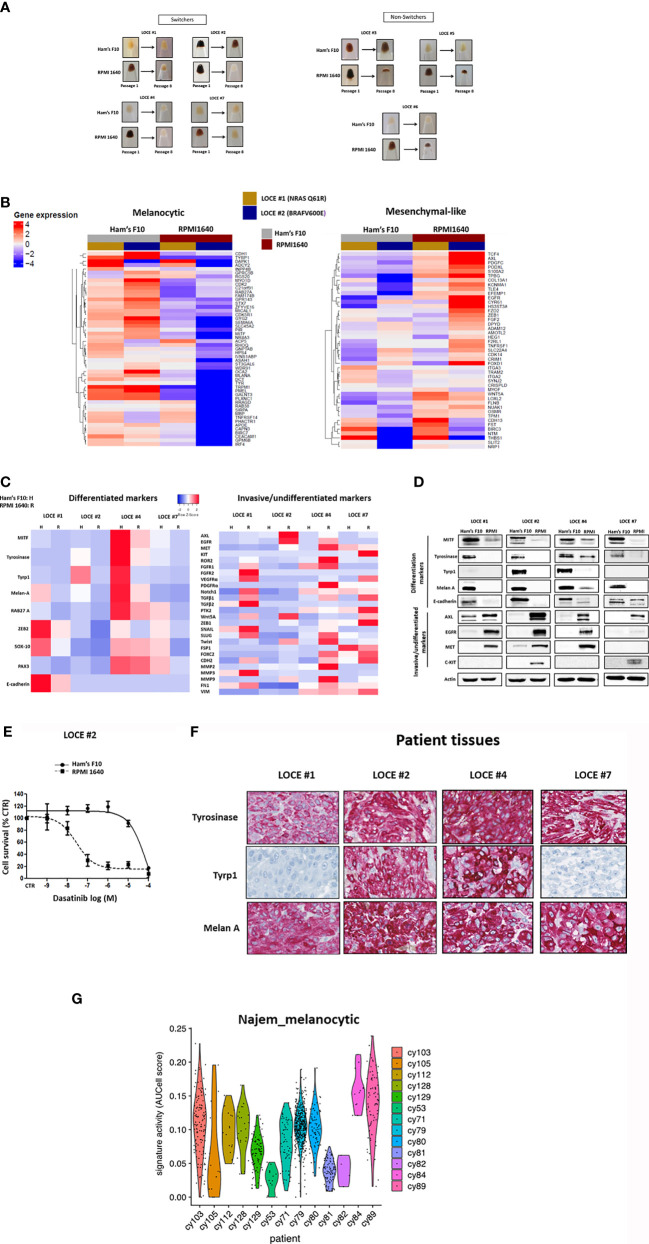
Growing primary melanoma cultures in low-Tyrosine medium allows the maintenance of a clinically-relevant highly differentiated melanoma state. **(A)** Comparison of visible pigmentation of cell pellets from four primary cultures (LOCE #1, 2, 4 and 7) (group of switchers) and three primary cultures (LOCE #3, 5, and 6) (group of non-switchers) established in 2 culture media (Ham’s F10 and RPM 1640) at passages 1 and 8. **(B)** Heatmap of genes in the Hoek signatures that are differentially expressed in the primary melanoma cultures (LOCE #1 and 2) at passage 8 for both the melanocytic and mesenchymal-like clusters in the two culture media (Ham’s F10 and RPMI 1640) measured by RNA-seq. **(C)** Heatmap of gene expression levels for representative markers of the differentiated and invasive states (rows) in the four pigmented primary cultures (LOCE #1, 2, 4 and 7) (switchers group) established in two culture media (Ham’s F10 and RPM 1640) at passage 8. **(D)** Comparative protein expression (representative Western blot) of main differentiation markers and RTK expression within the four pigmented primary cultures (LOCE #1, 2, 4 and 7) (switchers group) established in the 2 culture media (Ham’s F10 and RPM 1640) (passage 8). **(E)** Phenotype switching induced in RPMI 1640 leads to marked sensitivity to TKI (Dasatinib) showed in survival curves of switcher primary culture (LOCE #2) titrated with TKI (Dasatinib) for 72h. Data are shown as Means ± SEM (n=3) compared to untreated cells (Control, CTR). **(F)** IHC staining of melanocyte differentiation markers in paraffin sections of corresponding patient’s tumors tissues from where the four pigmented primary cultures (LOCE #1, 2, 4, and 7) (switchers group) are derived. **(G)** Melanocytic state activities in human treatment-naïve biopsies. Both melanocytic signatures were measured using AUCell (44) in scRNA-seq data of 13 human melanoma samples (44) and depicted as violin plots.

Similarly, to our previous observations using Ham’s F10 with 100 µM tyrosine, the cells growing in RPMI1640 could be rapidly grouped into 2 categories (**Figure 7A**). A first group of pigmented cells (termed switchers) (4/7) (LOCE #1, LOCE#2, LOCE #4, and LOCE #7) drifted towards an undifferentiated phenotype. In sharp contrast, the cells originating from the same tumors and cultured in Ham’s F10 medium maintained their differentiated phenotype. A second group of pigmented cells (termed non-switchers) (3/7) (LOCE #3, LOCE #5, and LOCE #6), became rapidly senescent and the corresponding cultures were lost (as documented by the very small cell pellets at passage 8 with residual cells) (**Figure 7A**). By contrast, in Ham’s F10 medium (low tyrosine) cells originating from the same tumor could be propagated for multiple passages.

RNA-seq analysis at passage eight of two of the switchers group (LOCE #1, NRAS Q61R and LOCE #2, BRAF V600E) revealed that the RPMI 1640 medium induces a decrease in expression of the differentiation gene signature and a concomitant increase in the invasive gene signature (**Figure 7B**). This was confirmed by RTqPCR focused on the main melanoma cell state markers in all four switcher primary cultures (LOCE #1, LOCE #2 LOCE #4, and LOCE #7) (**Figure 7C**). We observed various degrees of downregulation of melanocytic lineage markers such MITF, SOX10 and their downstream targets in these cells and upregulation of invasive markers such as RTKs, and other EMT markers including ZEB1 and MMPs (**Figure 7C**).

Consistently, a down-regulation of MITF, its downstream differentiation markers (Tyrosinase, Tyrp1 and Melan-A) and E- cadherin was observed at the protein level (**Figure 7D**). The decrease of such differentiation markers was associated with an upregulation/induction of RTK such as AXL, EGFR, MET and KIT (**Figure 7D**). Similar results were obtained with another rich culture medium (DMEM) which contains an even higher concentration of tyrosine (400 μM). We also found that DMEM promoted a phenotype switch from differentiated to undifferentiated/invasive phenotype (data not shown).

These data show that, similarly to Ham’s F10 medium supplemented with tyrosine to reach 100 µM, culture media such as RPMI1640 and DMEM promote dedifferentiation towards the mesenchymal-like state and strongly suggest that the effect observed in these culture conditions is caused by the high tyrosine concentrations.

Primary melanoma cultures are widely used models to screen novel therapeutic targets and strategies. Given the transcriptomic reprogramming observed in cells cultured in RPMI 1640 medium, we reasoned that this may affect the sensitivity of the cells to therapy. We compared the sensitivity to MAPK and RTK inhibitors of the four switchers in two distinct culture media, Ham’s F10 and RPMI 1640 (**Table 4**). All four primary cultures grown in RPMI exhibited a lower sensitivity to MAPKi and a significantly higher sensitivity to TKI (dasatinib and sunitinib). Such high sensitivity, in particular to dasatinib (**Figure 7E**), can be explained by the upregulation of various RTKs known as its targets (AXL, EGFR, c-Met, and c-Kit) (**Figures 7B**–**D**).

**Table 4:** Culture media composition influences the sensitivity to targeted drugs.

IC50 values (in micromolar)									
Primary cultures	Culture medium	BRAF inhibitor		MEK inhibitor		Tyrosine kinase inhibitors			
		Vemu	Ratio IC50 (H/R)	Pima	Ratio IC50 (H/R)	Dasa	Ratio IC50 (H/R)	Suni	Ratio IC50 (H/R)
LOCE #2 (BRAF V600E)	Ham’s F10	4.6	0.28	6.9	0.40	79.7	1328.33	29.3	24.42
	RPMI 1640	16.3		17.2		0.06		1.2	
LOCE #4 (BRAF V600E)	Ham’s F10	0.008	0.40	0.0009	0.90	52.8	1.20	11.1	3.58
	RPMI 1640	0.02		0.001		43.9		3.1	
LOCE #1 (NRAS Q61R)	Ham’s F10	18.1	0.77	0.001	0.33	3.9	3.55	9.3	4.89
	RPMI 1640	23.5		0.003		1.1		1.9	
LOCE #7 (NRAS Q61R)	Ham’s F10	36.4	0.88	0.001	0.25	5.1	1.59	60.2	8.36
	RPMI 1640	41.6		0.004		3.2		7.2	

IC50 mean values and ratios (Ham’s F10 (H)/RPMI-1640 (R)) obtained with BRAF inhibitor (Vemurafenib), MEK inhibitor (Pimasertib), and TKI (Dasatinib, Sunitinib) in the four pigmented primary cultures (LOCE #1, LOCE #2, LOCE #4 and LOCE #7) (switchers group) established in the 2 culture media (Ham’s F10 and RPM 1640).

These results clearly demonstrate that the choice of culture medium used to establish melanoma cultures can dramatically affect their sensitivity to anti-cancer drugs.

### Growing Primary Melanoma Cultures in Low Tyrosine Medium Allows the Maintenance of a Clinically Relevant Highly Differentiated Melanoma State

The corresponding patient’s tumor tissues from which the four primary melanoma cultures of the switcher group were derived expressed high levels of melanocyte lineage/differentiation markers (**Figure 7F**). Expression of these markers was maintained in cells cultured in Ham’s F10 medium with low tyrosine, showing that such cultures remain phenotypically close to the original tumor (**Figures 7D, F**). In contrast, these differentiation markers were lost or decreased in cells cultured in RPMI medium (and DMEM) (**Figure 7D** and data not shown).

Similarly, tumors at the origin of the non-switchers group of cells (LOCE #3, LOCE #5 and LOCE #6), also exhibited high expression levels of melanocyte lineage/differentiation markers and, again, expression of these markers was maintained in these three primary cultures grown in Ham’s F10 medium (**Supplementary Figures 1A, B**)

Finally, we derived a stringent gene expression signature of melanoma cells grown in low versus high tyrosine culture medium. We identified 78 over-expressed genes (log2FC > 4 and padj <0.0001) (**Table S1**). Among the top over-expressed genes, we detect genes implicated in cell differentiation (e.g. TMPRSS13, MAL, MLANA), melanocyte transcriptional programs (e.g. ST8SIA6, HTR2B, MX2, CA12), pigmentation (e.g. TYR, TYRP1, TRPM1, OCA2, S100B, APOD, CCL18, MLC1), retinal pigment epithelium (e.g. MAMDC2, TFPI2, ASPA, CDH3, SERPINF1), targets of MITF (e.g. PLA1A, CTSK) and of SOX10 (e.g. PMP2) [these target genes appeared in differential gene expression upon transfection with MITF or SOX10 (45, 46)] (**Table S2**). To further assess the translational relevance of our findings, we checked the existence of melanoma cells with activity of this stringent signature using the single-cell RNAseq data from drug-naïve melanoma cells (44). We identified a large number of positive cells in multiple biopsies (**Figure 7G**), indicating that such state is clinically relevant. Only 3 (cy53, cy81, cy82) out of 13 lesions did not show any cells in this melanocytic state.

These data highlight the importance of establishing melanoma cultures in low-tyrosine culture medium (Ham’s F10) in order to obtain cell cultures that maintain a highly differentiated/melanocytic state present in the tumor of origin.

## Discussion

Melanogenesis is activated by a multitude of stimuli that promote oxidative stress (15, 20, 24). It is also induced by the amino-acid tyrosine, which is the substrate of the key enzyme tyrosinase (17). Melanogenesis stimulation can affect cellular metabolism and nanomechanical properties, and thus melanoma phenotype (24, 47–49).

Our work stems from two observations that: 1) tyrosine level vary drastically between culture media commonly used for establishing and propagating melanoma cells, with RPMI 1640 being by far the most frequently used; and 2) pigmented human melanoma cultures are surprisingly rarely observed in contrast to human melanomas. A recent characterization of melanoma cultures established in our lab by (single-cell) RNA-seq identified however a high proportion of differentiated/melanocytic primary cultures (7). These observations prompted us to evaluate whether tyrosine content may influence differentiation status and thereby the value of these human primary melanoma cultures as a representative model for the human disease.

**Figure 8:**
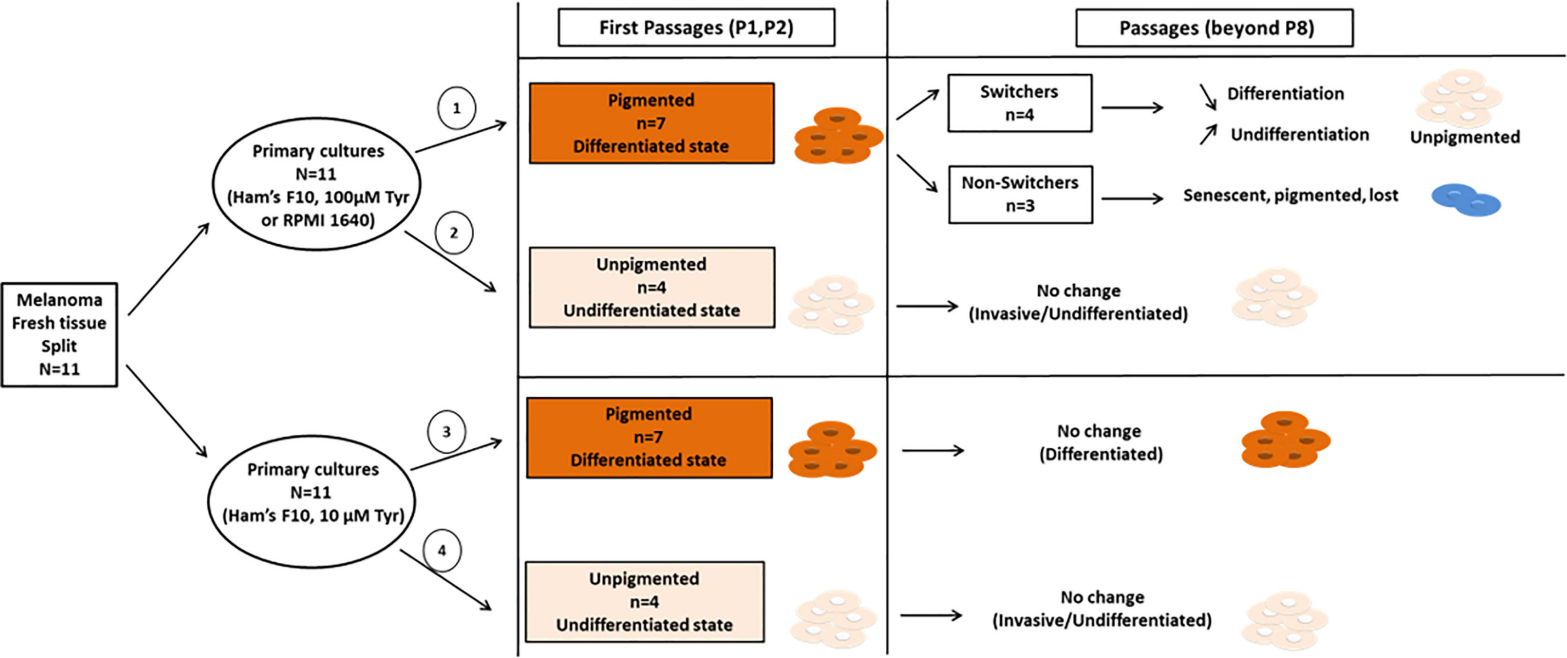
Schematic overview of the results presented in our study. Early primary cultures derived from eleven different patients were split and each primary culture from the same tissue was propagated in two different microenvironmental conditions with low tyrosine (Ham’s F10) or high tyrosine (Ham’s F10 supplemented with tyrosine; or RPMI 1640). Out of eleven primary cultures, seven initially displayed highly differentiated pigmented phenotype (cell pellets, passages 1-2), and four primary cultures displayed an invasive/undifferentiated unpigmented phenotype (cell pellets, passages 1-2). Row 1, High tyrosine medium (Ham’s F10 supplemented with tyrosine or RPMI 1640) promotes an early phenotypic switch (passage 8 and beyond) in originally primary cultures (n=7) towards an invasive/undifferentiated phenotype (switchers, n=4) or senescence-like phenotype (non-switchers, n=3). Row 2 and 4, The “unpigmented” primary culture group (n=4) presented at once with an inherent invasive/undifferentiated phenotype, with no phenotypic changes during passages, and independently of tyrosine content in culture media. Row 3, In low tyrosine culture medium, the pigmented cultures (n=7) did not switch or change pigmentation and have not been lost. These cultures keep their highly differentiated phenotype of the original tissues.

Out of eleven primary cultures established each from fresh melanoma tissues and propagated in parallel in three different media (33 primary cultures), seven originally (time zero) displayed a highly differentiated phenotype. High tyrosine exposure at early passage (P1, P2) rapidly caused/enhanced stimulation of pigmentation. The continuous exposure to high concentrations of tyrosine, and subsequent pigmentation stimulation, eventually led to a rapid loss of pigment and to a phenotypic drift towards a de-differentiated state in 4 out of 7 cultures (group termed switchers) (**Figure 8**). In the remaining cultures (3 out of 7) the persistence of high pigmentation levels promoted the entry into a senescence-like state (group termed non-switchers) (**Figure 8**). The latter primary cultures were completely lost between passages 8-10. None of these cultures drifted away from their original phenotype in low tyrosine medium (Ham’s F10) and could be maintained beyond passage 25. On the other hand, four “unpigmented” primary cultures keep their inherent undifferentiated phenotype independently of tyrosine content (**Figure 8**).

The switchers group presented a downregulation of melanocytic differentiation markers and an induction of RTKs and EMT markers. Similar phenotype switching events have previously been reported (1, 6–8, 11, 35) and can be caused by various tumor microenvironmental (TME) signals (9, 12–14) or therapeutic agents (3, 6, 11). We show herein that raising the concentration of one single amino acid, tyrosine, in the culture medium is sufficient to induce a phenotype switching, due to its implication in promoting melanogenesis and hence continuous oxidative stress.

The “non-switchers” group exhibited a senescence-like phenotype after prolonged exposure to a high level of tyrosine indicating that this particular form of cell cycle arrest is one adaptive mechanism adopted by melanoma cells to survive in hostile environments. This observation raises the possibility that this particular program may also be induced in response to therapeutic insult and thereby contribute to drug resistance (29, 36, 50, 51). Notably, tyrosine/DOPA have been considered as regulators of melanogenesis by promoting the synthesis of pigment-producing enzymes and MSH receptors (17). A constant stimulation of pigmentation may result in a decrease of cell viability (20) due to the production of toxic quinonic and indolic derivatives that are melanogenesis intermediates and, most importantly, as a consequence of the intracellular accumulation of melanosomes, which cannot be transferred to surrounding cells in isolated cell cultures (15).

Analogously, the de-differentiation and differentiation trajectories followed respectively by switchers and non-switchers cultures are reminiscent to the cellular response observed in patient-derived xenograft (PDX) melanoma lesions following exposure of targeted therapy (3).

Our data point to an important role of ROS in the phenotypic plasticity of melanoma. Indeed, our findings, in line with previous studies (52, 53), identify that an increase in ROS levels is the main trigger underlying emergence of the invasive (EMT-like) phenotype and that these phenotypic adaptation mechanisms are ROS-dependent.

We also identified putative roles for RTKs and the cAMP-PKA axis in the regulation of phenotypic plasticity in melanoma. This is consistent with previous studies demonstrating that cAMP/PKA activation can reverse the EMT phenotype in mesenchymal human mammary epithelial cells (54) and that RTK signaling is linked to EMT and contributes to therapy resistance in several epithelial cancers (32, 35, 55).

Notably, high tyrosine concentration did not cause any phenotypic drift in the “unpigmented” primary culture group. Regardless of the presence of tyrosine, these cultures displayed an EMT-like phenotype and intrinsic resistance to MAPK inhibitors, which was maintained over passages, and showed vulnerability to TKI. TKI also inhibited their cell migration and invasiveness, an effect that was greatly enhanced by cAMP. These data further confirm that RTK and cAMP/PKA signaling have antagonistic functional roles in regulating the invasive melanoma phenotype.

More importantly, the comparison between pigmented and unpigmented groups of primary cultures regardless of their BRAF and NRAS status indicate that pigmented cultures displayed a differentiated phenotype while unpigmented cultures showed an undifferentiated/invasive phenotype. The relationship between pigmentation and cell phenotype is not well understood but recent studies demonstrated that melanin granules inhibit the invasive abilities of melanoma cells *in vitro* and melanoma cell spreading in mice (56, 57). Here we provide an experimental evidence in terms of phenotypic analysis at transcriptional as well as functional levels, that support the assumption that amelanotic melanomas are more aggressive than their pigmented counterparts.

Of note, the same results were obtained when using a rich tyrosine culture medium such as RPMI1640, commonly used to establish human melanoma primary cultures. Pigmented cell cultures either switched to a less differentiated phenotype or were lost. This could explain our high efficiency in establishing primary cultures maintaining similar characteristics to those of original tumors by using the low tyrosine culture medium Ham’s F10.

Recently, a revised rheostat model for the classification of phenotypic states has been proposed by Rambow et al. (1) in which six melanoma subtypes are identified based on their varying degree of differentiation: the hyperdifferentiated (pigmented), the melanocytic (corresponding to Hoek’s proliferative state and Tsoi’s C4 subtype) (11, 31), the intermediate (corresponding to Tsoi’s C3) (7, 11), the starved (3), the neural crest-like (corresponding to Tsoi’s C2) (3, 11) and the undifferentiated subtypes (Hoek’s invasive state and Tsoi’s C1) (11, 31). In the present study, we show that the majority of primary cultures in Ham’s F10 maintain a hyperdifferentiated/melanocytic phenotype while other primary cultures have an inherent invasive phenotype. In contrast, we show that the same differentiated cultures, grown in tyrosine-rich media such as RPMI1640, can undergo an early transition towards a less differentiated state, and thereby loose the differentiated phenotype present in their tumor of origin. As consistent with previous findings (58, 59), we show here in that the majority of the melanoma cells present in tumor biopsies display a differentiated phenotype.

Altogether, we show that primary cultures established in high tyrosine media rapidly become less differentiated, more invasive but also retain high proliferation capacity. Likewise, melanoma lines frequently used worldwide such as SKMEL 5, SKMEL 28 and M14 are considered as melanocytic, but in fact are amelanotic (60, 61). Moreover, melanocytic lines such as SKMEL28, WM9 and MEL501 show considerable invasive activity in several studies (62–64). Other melanocytic cell lines such as M202, M207 and M249 have a very short doubling time (approximately 24 hours) (65), reflecting a less differentiated and more proliferative state. All these lines are propagated in RPMI culture medium.

Our results may also explain the low success rate during establishment of primary melanoma cultures in RPMI medium (about 45%) (66) because of the possible loss of cultures through the activation of a senescence process.

In conclusion, our data demonstrate that tyrosine promotes an early phenotypic switch in differentiated human melanoma cells towards a less differentiated phenotype or senescence-like phenotype and hence greatly affects the establishment of these melanoma primary cultures and their clinical relevance.

## Data Availability Statement

The RNAseq data have been deposited in the GEO repository under accession number GSE171456.

## Ethics Statement

Written informed consent was obtained from the individual(s) for the publication of any potentially identifiable images or data included in this article.

## Author Contributions

AN and GG conceived the research study and designed the experiments. AN performed the *in vitro* phenotypic assays, analyzed, and interpreted the data with MK, MS, and FJ. JW and SA performed RNA sequencing experiments. JW, FR, SA, and J-CM performed bioinformatics analyses and contribute to data interpretation. FS and AA provided patient tumor samples and contributed to the manuscript. AN, GG and J-CM wrote the manuscript with input from all authors. All authors approved the submitted version.

## Funding

The work has been supported by grants from the ‘Amis de l’institut Bordet’, Fondation Lambeau-Marteaux (to AN), postdoctoral research fellowship from Kom op tegen Kanker (Stand up to Cancer), the Flemish Cancer Society and from Stichting tegen Kanker (Foundation against Cancer), the Belgian Cancer Society (to JW).

## Conflict of Interest

The authors declare that the research was conducted in the absence of any commercial or financial relationships that could be construed as a potential conflict of interest.

## Publisher’s Note

All claims expressed in this article are solely those of the authors and do not necessarily represent those of their affiliated organizations, or those of the publisher, the editors and the reviewers. Any product that may be evaluated in this article, or claim that may be made by its manufacturer, is not guaranteed or endorsed by the publisher.
