# Supplementary material for: Tyrosine-Dependent Phenotype Switching Occurs Early in Many Primary Melanoma Cultures Limiting Their Translational Value
Source: Front Oncol. 2021 Nov 11;11:780654. doi: 10.3389/fonc.2021.780654 (PMC8635994; doi:10.3389/fonc.2021.780654)
Supplement: Supplementary file 1 [file DataSheet_1.docx]

Supplementary Material


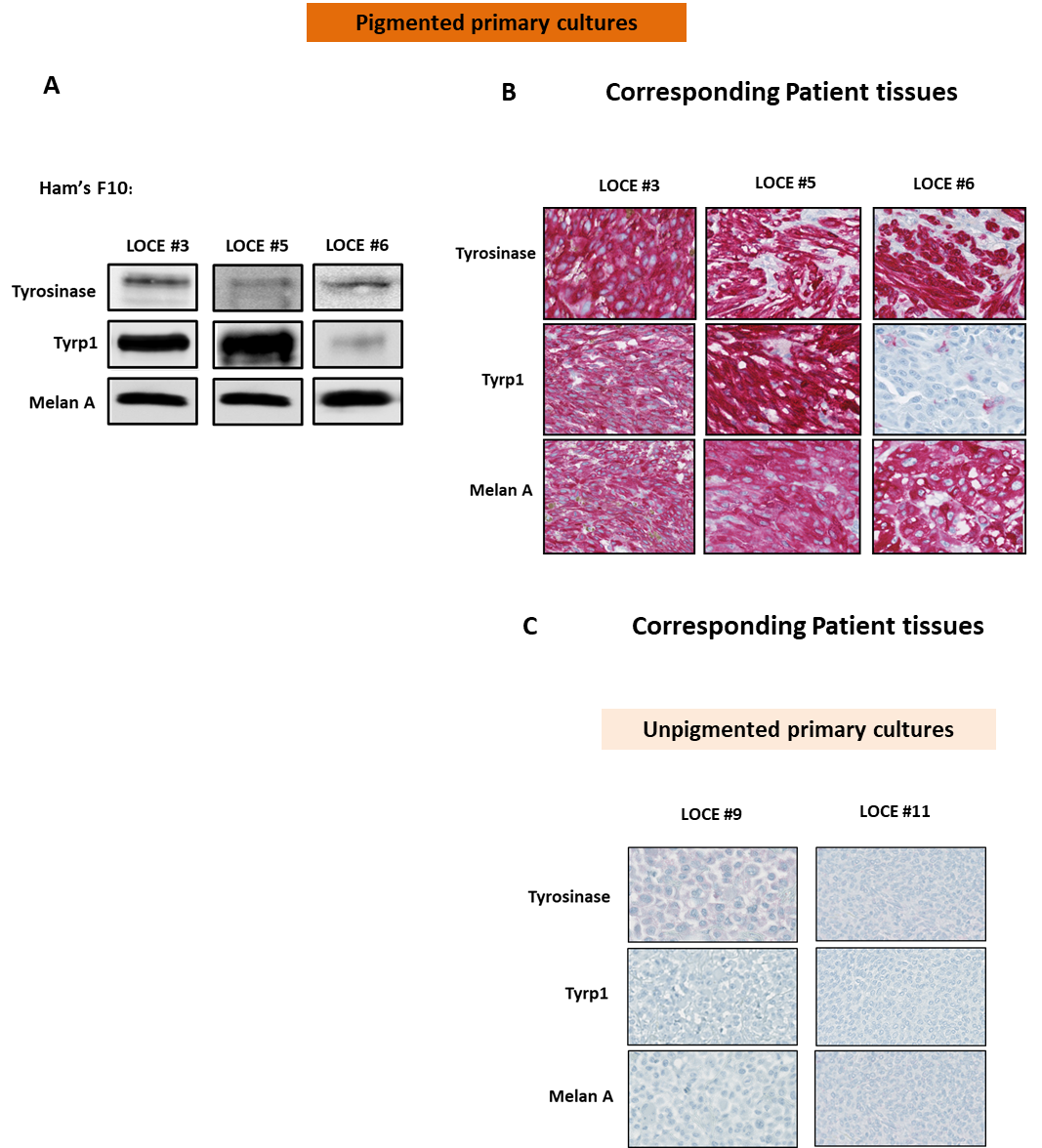


Supplementary Figure 1. (A) Protein expression (representative western blot) of main differentiation markers (β actin served as a loading control) in three primary cultures (LOCE #3, 5, and 6) (group of non-switchers) established in low-tyrosine medium (Ham’s F10). (B and C) IHC staining of melanocyte differentiation markers in paraffin sections of corresponding patient’s tumors tissues from where the three pigmented primary cultures (LOCE #3, 5, and 6) (non-switchers group) and two unpigmented cultures (LOCE #9 and 11) are derived.

Table S1. List of 78 genes differentially expressed (log2FC > 4 and padj <0.0001).

Table S2. The Top 35 differentially expressed Genes and their relevance in cell differentiation and pigmentation.

Table S3. Differential expression results of the RNA-seq data. Differential expression between Ham’s F10, Ham’s F10-tyrosine and RPMI 1640 samples (log2FC, p-value and adj. p-value).

Table S4. Characteristics of tissues used for the establishment of melanoma primary cultures.
