# Supplementary material for: Tyrosine-Dependent Phenotype Switching Occurs Early in Many Primary Melanoma Cultures Limiting Their Translational Value
Source: Front Oncol. 2021 Nov 11;11:780654. doi: 10.3389/fonc.2021.780654 (PMC8635994; doi:10.3389/fonc.2021.780654)
Supplement: Supplementary file 3 [file Table_2.docx]

| **Top 35 differentially expressed Genes** | **Function (Relevance in cell differentiation and pigmentation** |
| --- | --- |
| TMPRSS13 | Epidermal differentiation (Madsen et al., 2014) |
| MAMDC2 (MAM Domain Containing 2) | Identified in the molecular signature of pigmented (PE) neuro-epithelia of the ciliary body (Janssen et al., 2012) |
| CA12 (Carbonic Anhydrase 12) | Melanocyte differentiation (Raja et al., 2020) via PH regulation |
| CCL18 | Identified as one of the top upregulated gene in Hyperpigmented Skin (Yin et al., 2015) |
| SERPING1 | Retinal pigment epithelium (RPE) (Klaver & Bergen, 2008) |
| TFPI2 (Tissue Factor Pathway Inhibitor 2) | Retinal Pigment Epithelium Cell Factor (Obata et al., 2005; Shibuya et al., 2007) |
| MLC1 (Modulator Of VRAC Current 1) | - Key roles in the regulation of neuronal, glial and vascular homeostasis (Hwang et al., 2019) - intracellular trafficking through early endosomes (Brignone et al., 2014) - Difference in pigmentation Between Drosophila (Carbone et al., 2005) |
| Aspa (Aspartoacylase) | Abnormal retinal pigmentation phenotype (HPO Gene-Disease Associations dataset) |
| PLA1A (phospholipase A1 member A) | MITF target whose expression was increased by at least 50‐fold upon Mitf transfection (Hoek et al., 2008) |
| APOD (Apolipoprotein D) | - Potential role in carotenoid-based pigment in birds (GAO et al., 2016). - Expansion of the apolipoprotein D (ApoD) ligand-transporter genes in a cluster manner specific to teleost fishes (Gu & Xia, 2019). |
| MAL (Mal, T Cell Differentiation Protein) | Strong relation between differentiation status and MAL expression (Liebert et al., 1997; Wakeman et al., 1997) |
| PMP2 (peripheral myelin protein 2) | SOX10 target , identified among most differentially regulated genes and consistently upregulated in several human melanoma lines after ectopic expression of SOX10 (Graf et al., 2019) |
| MX2 (myxovirus resistance 2) | High expression of MX2 RNA in melanocytes and primary melanoma tumors (Choi et al., 2020; Juraleviciute et al., 2020) |
| TMEM255A (transmembrane protein 255A) | proteins of this family are predicted to be components of various organelles membranes (Schmit & Michiels, 2018) |
| CDH3 ( cadherin-3 (also called P-cadherin) | Loss of E-cadherin (CDH1), P-cadherin (CDH3) or both during tumour progression results in an increased expression of the mesenchymal cadherin,(Ciołczyk-Wierzbicka & Laidler, 2018)  expressed in human retinal pigment epithelium (RPE) and critical mediator of cell-cell adhesion3 which is) (Singh et al., 2016) |
| S100B (S100 calcium-binding protein B) | Pigment-related protein and exhibit high positive rates in MM and pigment disorders (J. Xia et al., 2016) |
| ST8SIA6 (Alpha-N-Acetyl-Neuraminide Alpha-2,8-Sialyltransferase 6) | expressed at a higher level in melanocytes (Reemann et al., 2014) |
| CTSK (Cathepsin K) | Identified as MITF target upon transformation with a MITF-expressing vector (P < 0.05). (Hoek et al., 2008) |
| METTL7A( Methyltransferase like 7A) | Transformation from melanocyte to melanoma (Hoek, 2007) |
| HTR2B (serotonin receptor, 5-hydroxytryptamine receptor 2B) | Highly expressed in melanocytes (transcriptome study) (Haltaufderhyde & Oancea, 2014) |
| PDE3A (Phosphodiesterase 3A) | identification of phosphodiesterase 4D3 as a direct target of the MSH/cAMP/MITF pathway (Khaled et al., 2010) |
| OCA2 (oculocutaneous albinism II) | integral membrane melanocyte-specific transporter protein , involved in small molecule transport, specifically of tyrosine (Toyofuku et al., 2002) |
| EMILIN1 (Elastin Microfibril Interfacer 1) | located in the dermis, up to the basement membrane, and interacts with components of the extracellular matrix (Fitoussi et al., 2019) |
| MPZ (Myelin Protein Zero) | SOX10 target (LeBlanc et al., 2006) |
| CHSY3 Chondroitin Sulfate Synthase 3) | MITF target (M. Xia et al., 2017) |
| SLC22A17 (Solute Carrier Family 22 Member 17) | SLC22A17 was detected in human RPE (Parmar et al., 2018) |
| ALDH1A1 (Aldehyde Dehydrogenase 1 Family Member A1) | ALDH1A1 substrates potently induce the accumulation of MITF and tyrosinase mRNA  (Kleszczynski & Slominski, 2013; Paterson et al., 2013) |
| LINC00589 (Long Intergenic Non-Protein Coding RNA 589) | MITF target (CHEA Transcription Factor Binding Site Dataset) |
| PRELP( Proline And Arginine Rich End Leucine Rich Repeat Protein) | MITF target (CHEA Transcription Factor Binding Site Dataset) |
| FXYD3 (FXYD Domain Containing Ion Transport Regulator 3) | The retinal pigment epithelium (Cowan et al., 2019) |
| SERPINF1 (Serpin Family F Member 1) | Pigment epithelium-derived factor (PEDF) |
| TYRP1, TRPM1, MLANA | Melanocyte specific genes |
| CRYAB (Crystallin Alpha B) | CRYAB is secreted from human retinal pigment epithelial cells (D’Agostino et al., 2019) |
