# Supplementary material for: Tyrosine-Dependent Phenotype Switching Occurs Early in Many Primary Melanoma Cultures Limiting Their Translational Value
Source: Front Oncol. 2021 Nov 11;11:780654. doi: 10.3389/fonc.2021.780654 (PMC8635994; doi:10.3389/fonc.2021.780654)
Supplement: Supplementary file 5 [file Table_4.docx]

| Primary cultures | Melanoma type | Gender | BRAF  status | NRAS status | TP53 status | Metastatic site | Pigmentation |
| --- | --- | --- | --- | --- | --- | --- | --- |
| LOCE#1 | NM | F | WT | NRAS Q61R | ND | LN | - |
| LOCE#2 | NM | F | BRAF V600E | WT | ND | LN | + |
| LOCE#3 | ALM | F | WT | WT | WT | LN | + |
| LOCE#4 | SSM | F | BRAF V600E | WT | V272M | LN | + |
| LOCE#5 | SSM | M | WT | NRAS Q61R | ND | LN | - |
| LOCE#6 | SSM | F | BRAF V600E | WT | ND | LN | + |
| LOCE#7 | SSM | M | WT | NRAS Q61R | P72R | SK | + |
| LOCE#8 | SSM | M | WT | NRAS Q61K | WT | LN | - |
| LOCE#9 | SSM | M | BRAF V600K | WT | WT | LN | - |
| LOCE#10 | SSM | M | WT | NRAS Q61R | ND | SK | - |
| LOCE#11 | SSM | F | BRAF V600E | WT | WT | SK | - |

SSM: superficial-spreading melanoma; ALM: acral lentiginous melanoma; NM: nodular melanoma; F: female; M: male, WT: wild type; ND: Not done; LN=lymph node, SK=skin/cutaneous metastasis
